# Diagnostic Challenges of *Helicobacter pylori* Infection in Ethiopia: A Community-Based Cross-Sectional Study

**DOI:** 10.1155/2022/4013020

**Published:** 2022-06-02

**Authors:** Esayas Kebede Gudina, Hiwot Amare, Solomon Ali, Melkamu Berhane Arefayine, Dagmawi Tewolde, Million Tesfaye Eshete, Mulusew Gerbaba Jebena, Andreas Wieser, Guenter Froeschl, Markos Tesfaye, Hailemichael Desalegn, Mulatu Gashaw

**Affiliations:** ^1^Department of Internal Medicine, Jimma University, Jimma, Ethiopia; ^2^Department of Microbiology, St. Paul's Hospital Millennium Medical College, Addis Ababa, Ethiopia; ^3^Department of Pediatrics, Jimma University, Jimma, Ethiopia; ^4^Department of Anesthesiology, Jimma University, Jimma, Ethiopia; ^5^Department of Epidemiology, Jimma University, Jimma, Ethiopia; ^6^Division of Infectious Diseases and Tropical Medicine, University Hospital, Ludwig Maximilian University of Munich, Munich, Germany; ^7^German Center for Infection Research (DZIF), Partner Site Munich, Munich, Germany; ^8^Department of Psychiatry, St. Paul's Hospital Millennium Medical College, Addis Ababa, Ethiopia; ^9^Department of Internal Medicine, St. Paul's Hospital Millennium Medical College, Addis Ababa, Ethiopia; ^10^School of Medical Laboratory Sciences, Jimma University, Jimma, Ethiopia

## Abstract

**Background:**

In resource-constrained countries, accurate diagnosis of *Helicobacter pylori* infection remains a challenge. This study aimed to assess the clinical utility of locally available serological and stool antigen test kits in the management of people with suspected *H. pylori* infection in Ethiopia.

**Methods:**

A community-based cross-sectional study was conducted with apparently healthy adults and children living in southwest Ethiopia. Participants were interviewed for dyspepsia symptoms and related clinical conditions. *H. pylori* infection was examined using commercially available serological and stool antigen tests. The association between *H. pylori* tests and dyspepsia symptoms was analyzed using logistic regression models.

**Results:**

Out of 1392 participants included in the final analysis, 49.1% and 6.5% tested positive for *H. pylori* infection with serology and stool antigen test kits, respectively. Participants reporting epigastric symptoms in the past three months (AOR = 1.93, 95% CI = 1.28–2.91) and those with recent dyspepsia treatment (AOR = 1.51, 95% CI = 1.05–2.18) were likely to have positive serology test. However, no association between dyspepsia symptoms and *H. pylori* stool antigen positivity was observed in our study.

**Conclusion:**

ccurate detection of *H. pylori* infections using commercially accessible diagnostics remains difficult in Ethiopia. With these methods, it will be hard to ensure adequate diagnosis and early treatment of *H. pylori* infection, as well as rational antibiotic use.

## 1. Introduction

*Helicobacter pylori* (*H. pylori*) has become a pathogen with significant public health importance because of its association with multiple malignant and nonmalignant disorders [1]. It is known to cause functional dyspepsia, peptic ulcer disease, gastric cancer [2], and gastric mucosal-associated T-cell lymphoma [3]. In children, *H. pylori* infection is associated with growth faltering [4] and iron deficiency anemia [5].

*H. pylori* infection is usually acquired in early childhood through intrafamilial transmission or acquisition from environmental sources [6]. Unless eradicated by combined antibiotic therapy or cleared by the immune response, the infection can persist for life [7]. As a result, *H. pylori* infection has become an endemic disease all over the world. The real prevalence of the infection has, in fact, become an indicator of socioeconomic status of the countries and hygiene standards of the settings. While there is a significant reduction in the incidence of infection among children in high income countries over the last few decades, the burden in most of low- and middle-income countries remains very high [8]. The current global prevalence of *H. pylori* infection varies from 20 to 50% in industrialized countries [9] to about 80% in the developing world [6, 10].

Treatment of *H. pylori* infection is of paramount importance for the prevention and management of both nonmalignant and malignant disorders associated with this pathogen [11]. Most countries with high prevalence of *H. pylori* infection implement a “test-and-treat” strategy in patients with dyspepsia to reduce the risk of ulcers and gastric cancer [12, 13]. However, there are concerns regarding the universal applicability of this strategy in the general population due to the risk of antibiotic resistance [14]. Moreover, the availability, validity, cost-effectiveness, and applicability of diagnostic tests for the screening of the infection vary in different geographic and population settings [12]. In resource-limited countries, there is a paucity of data on the validity of available tests for *H. pylori* infection, and no clear guideline or consensus is available for “test-and-treat” strategy.

Urea breath test is regarded as a gold-standard noninvasive test to diagnose *H. pylori* infection [15] and a test of choice to assess response to eradication therapy [16]. Invasive diagnostic methods that involve endoscopy evaluation and collection of gastric biopsies provide more comprehensive evaluation through rapid urease test, histopathology, culture, or molecular tests [17]. However, both these noninvasive and invasive methods are expensive and often not available in public healthcare facilities in most low-income settings such as Ethiopia. As a result, cost-effective testing strategies and treatment approaches applicable in most low-income and high *H. pylori* prevalence settings are not established. As both *H. pylori* serology and stool antigen tests become increasingly available in low-income settings, it is important to determine the challenges and limitations of these assays as an element for treatment decisions. For example, with serology, it is impossible to distinguish between current infection and previous exposure [17]. On the other hand, stool antigen test is suitable for identifying the current active infection, but diagnostic sensitivity remains a challenge. In addition, low-resource countries like Ethiopia are forced to heavily rely on low-cost manufacturers that frequently lack compliance with the requirements of validation and good manufacturing practices, which increases the difficulties in diagnosing *H. pylori* infection in low-income countries [18]. However, on the ground, clinicians at the forefront of primary care do not dispose of alternatives to these assays, and both false positive and false negative diagnoses may lead to antibiotic misuse and risk of antimicrobial resistance [19]. Therefore, the main goal of this study was to assess the clinical utility of these tests in the management of people with suspected *H. pylori* infection in Ethiopia.

## 2. Methods and Materials

### 2.1. Study Design and Setting

A community-based cross-sectional study was conducted in Jimma Zone, southwest Ethiopia, from September to October 2019.

### 2.2. Sample Size Estimation

The number of study participants was determined based on the risk of H. pylori infection. Accordingly, participants were classified into five age categories: (1) 1–5, (2) 6–10, (3) 11–19, (4) 20–29, and (5) ≥30 years. The sample size was calculated for each of these categories using Epi Info™ Version 7.2.4.0 using the sample size formula for the population survey. Assumptions taken into account to calculate the sample size were a population size of >10000 for each category; 5% margin of error; and an expected proportion of *H. pylori* infection among non-symptomatic population of 41% for under-five [20], 10% for school age [20], and 52% for the remaining age groups [21]. Finally, a total of 1422 study participants were recruited.

### 2.3. Sampling and Participant Selection

Representative samples of adults and children were included through multistage and cluster sampling techniques. Nine districts (woreda), one urban (Jimma Town) and eight rural districts, were randomly selected. All woredas were allocated about 10.8% of the total sample size each, except Jimma Town which was allocated 14%. Three kebeles were randomly selected from each district except Jimma Town where four kebeles were selected due to its high population size. Finally, fifty households (HH) were randomly selected in each kebele.

The first HH was randomly selected from the lists of households registered by health extension workers in each kebele. Once the first household was selected, the next HH at a walking distance was approached and assessed for eligibility. This was continued until the final sample size was achieved. Due to intracluster correlation within a household, we used parallel sampling techniques for adults, adolescents, and child survey. If the selected family had more than one eligible person, a lottery method was used to select one study participant from each HH. Only apparently and self-declared healthy individuals who gave consent and were willing to provide both blood and stool samples were recruited. Individuals who consented to participate in the study but were unable to give blood and/or stool specimens at the time of data collection or whose specimens had to be discarded for technical reasons afterwards were excluded from the final analysis.

### 2.4. Data Collection Procedures

All eligible participants were interviewed for symptoms of dyspepsia and related gastrointestinal conditions. Dyspepsia in this study was defined if the participant reported the occurrence of one or more of the following symptoms persisting for the past three months: epigastric pain, epigastric discomfort, epigastric burning, postprandial fullness, early satiation, abdominal bloating, heartburn, acid regurgitation, and excessive belching. Blood (3–5 ml) and stool samples were collected from each participant. The samples were evaluated following the standard operating procedures used at Jimma Medical Center (JMC) laboratory and manufacturer's recommendations to detect *H. pylori* infection.

For *H. pylori* antibody test, 3–5 ml of blood sample was collected from each participant in EDTA anticoagulated tube. The specimens were then stored in the refrigerator at 2–8°C and transported to JMC laboratory within eight hours of the collection. The antibody test was performed using Coretests® ONE STEP TEST KIT (Core Technology Co., Ltd., Beijing, China).

For stool antigen test, about 0.5 gram stool specimen was collected in a dry and clean container to test for the presence of *H. pylori* antigen. Stool antigen test was performed at the site using Wondfo® One Step H. Pylori Antigen Feces Test Cassette (Guangzhou Wondfo® Biotech Co., Ltd., Guangzhou, China) according to the manufacturer's instructions.

### 2.5. Data Analysis

The collected data was cleaned, entered into EpiData Entry version 3.1, and exported to SPSS® Statistics version 25 (IBM®, New York, USA) and Microsoft Excel (Microsoft, Redmond, WA, USA) for analysis.

Descriptive statistics tabulated for the total population and stratified based on test results for both serology and stool antigen were used to summarize the characteristics of the study participants. Logistic regression analysis was performed to determine the strength of associations between the symptoms of dyspepsia and the results of the two laboratory tests. Characteristics that showed an association in bivariate analysis (*P* < 0.20) were included in the multivariable logistic regression model. *P* value of 0.05 was used as a threshold of statistical significance.

### 2.6. Ethical Considerations

The study was approved by the Institutional Review Board of Jimma University Institute of Health (reference letter: IHRPGD/3012/18) and Oromia Regional Health Bureau. Written informed consent was obtained from each adult study participant (≥ 18 years of age). In case of children, <18 years of age, the consent of the guardian was collected, and additionally assent was obtained for adolescent minors (aged 12–17 years).

## 3. Results

### 3.1. Background Characteristics

A total of 1422 participants were included in the study. However, 30 (2.1%) participants with incomplete data were excluded, leaving 1392 individuals for the final analysis. About 57% were female participants. The median age was 19.00 (IQR = 6–30) years with a range of 1 to 80 years; a quarter of the participants (344, 24.7%) were between one and five years of age. Most of the participants (1198, 86.1%) were rural residents (Table 1).

### 3.2. Test Results for *H. pylori* Infection

**Figure 1:**
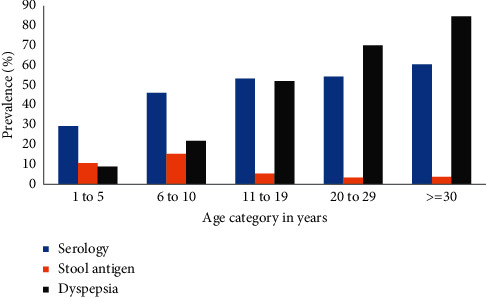
Prevalence of *H pylori* infection, by serology and stool antigen test, and symptoms of dyspepsia by age group. “Dyspepsia” on the graph refers to the presence of one or more of the dyspepsia symptoms.

The test positivity for *H. pylori* infection by serology and stool antigen tests was found to be 49.1% and 6.5%, respectively. However, significant differences were observed between the different age groups, both for serology and stool antigen tests. Seroprevalence of *H. pylori* increased with age from 29.4% in ≤5 years to 60.5% in ≥30 years. However, *H. pylori* stool antigen positivity was found to be highest during school age (6–10 years) with a prevalence of 15.4%; it declined with age to less than 5% in adults aged 20 years and older (Figure 1).

### 3.3. Symptoms of Dyspepsia

Overall, 723 (51.9%) of the participants reported one or more symptoms of dyspepsia in the preceding three months of the survey. Epigastric discomfort was the most common symptom reported in 592 (42.5%) of the participants. Excessive belching (418, 30.0%), early satiety (411, 29.5%), and postprandial fullness (379, 27.2%) were the other commonly reported symptoms of dyspepsia (Table 2). However, significant variability of all these symptoms was observed among age groups, being more common in adults of 30 years and older. At least half of the adults in this category reported almost all symptoms of dyspepsia in the three months preceding the data collection. However, dyspepsia symptoms were reported less often in children ≤10 years of age (Figure 1).

### 3.4. Association between Symptoms of Dyspepsia and *H. pylori* Test Results

A direct relationship between symptoms of dyspepsia and *H. pylori* seropositivity was observed on bivariate analysis. *H. pylori* serology was found to be positive in 58.3% of individuals with one or more symptoms of dyspepsia. Separate analysis for each dyspepsia symptom has shown *H. pylori* seropositivity rate of 57.5% to 61.2% compared to 40.1% to 46.7% among those with no symptoms (*P* < 0.001). Similarly, previous treatment (*P* < 0.001) and help-seeking for symptoms of dyspepsia (*P* < 0.001) were associated with seropositivity. Besides, 63.2% (43/68) of patients who were given a clinical diagnosis of stomach ulcer by their physician had a positive serology test compared to 48.3% (640/1324) in the other groups (*P* < 0.001). On the contrary, an inverse association between dyspepsia symptoms and stool antigen positivity was observed (Table 2). On multivariate logistic regression analysis, only epigastric burning in the past three months (AOR = 1.93, 95% CI = 1.28; 2.91) and treatment for dyspepsia in the past 30 days (AOR = 1.51, 95% CI = 1.05; 2.18) were associated with positive *H. pylori* serology (Table 3).

## 4. Discussion

Nearly half of the participants had positive *H. pylori* serology. However, the yield for stool antigen was found to be much lower than the expected prevalence for the setting. These findings correspond to the test characteristics, where serology can be seen as a cumulative indicator of past exposure, while stool antigen testing gives positive results only in case of current active infection. This also explains the age-stratified findings, where consistent variability with age was observed for both tests; while serology showed an incremental pattern, stool antigen declined with age.

Over half of the participants reported at least one symptom of dyspepsia in the preceding three months. Positive *H. pylori* serology showed a good correlation with symptoms of dyspepsia, which was not the case for the results of the stool antigen test, possibly showing the reported dyspepsia to be more a sign of chronic infection. Dyspepsia is a common condition, affecting 7–45% of people globally [22]. However, the prevalence of this symptom varies in different settings based on lifestyle, nutritional pattern, and definitions used [23, 24]. Moreover, countries with a known higher prevalence of *H. pylori* infection are likely to have higher rates of upper gastrointestinal symptoms [23]. In our study, 52% of the participants reported at least one symptom of dyspepsia and nearly 43% had epigastric symptoms in the preceding three months prior to data collection. The proportion of the participants with dyspepsia showed an age-related incremental pattern which is expected due to changes in lifestyle during adulthood [25]. This proportion is much higher than reports from previous community-based studies [22–24]. However, most of these studies were limited to Europe and North America, and hence comparison with our findings may suffer from host- and setting-specific confounders.

A high rate of *H. pylori* seropositivity was observed in this study among individuals who had recent symptoms of dyspepsia, who sought healthcare for their symptoms, and those who have recently received (proton pump inhibitors) PPIs. In contrary, an inverse association between these symptoms and stool antigen positivity was observed in bivariate analysis. While *H. pylori* antibodies are likely to persist for months or years after the bacteria are effectively eradicated by combination therapy [17, 26], stool antigen assays suffer from limited sensitivity as they may yield false negative results in patients who received PPI or antibiotics in the preceding month [17]. As a result, active *H. pylori* infection may be overdiagnosed if serology is used and can be missed if stool antigen is used as a primary diagnostic test.

The prevalence of *H. pylori* infection in low-income settings is highly variable with regard to differences in study settings, study designs, and types of tests used [9, 27]. Nevertheless, there is robust evidence of a very high disease prevalence in low- and middle-income countries [9, 28]. In this regard, the prevalence of 49.1% of positivity in our study is lower than what has been reported from Africa and other low-income settings [9]. However, our study included both children and adults, unlike most of the other studies, and hence is difficult to compare with studies that included adults only. Separate analysis for adults aged ≥20 years showed a prevalence of 57.5%, a figure comparable to the finding of a systematic review of studies from Ethiopia [21].

A consistent increment in *H. pylori* antibody seroprevalence with age was observed in our study. This pattern aligns with the fact that exposure to the pathogen is accumulated with age in poor living conditions [29]. Acquisition of *H. pylori* infection starts during early childhood [6] and continues throughout life in poor socioeconomic settings [30]. Unless it is eradicated by antibiotic therapy or cleared by the immune response [7], the infection may persist for life. Thus, an incremental change of disease prevalence with age, when assessed by serology studies, in particular, is expected for such infections.

Stool antigen positivity rate on the other hand showed a decline with age and was highest during school age. It may be due to the acquisition of the bacteria starting during early childhood [6]. The risk of infection intensifies during school age because of the child's increased exposure to the pathogen through the fecal–oral, oral–oral, (dental), and gastro–oral routes associated with poor hygiene facilities and practices as they are found outside the home environment, as, for example, in school facilities [31, 32]. This peak in acquisition of *H. pylori* infection is mirrored by higher detection of *H. pylori* antigen. However, with growing age from school age towards adolescence, there is an improvement in self-hygiene. Moreover, adolescents and adults are likely to be treated for symptoms of dyspepsia with medications such as PPIs, which may affect the bacterial load in the gastrointestinal tract and consequently the antigen load in stool. Likewise, an increased intake of antibiotics in older children and adults for various reasons results in a decline in bacterial load leading to a false negative test for stool antigen [33, 34]. Therefore, with increasing age, a shift can be expected from *H. pylori* antigen positivity towards serum antibody positivity, as the latter corresponds to an increased cumulative risk over the lifetime of having been exposed to the bacteria.

Despite these conceptual explanations, *H. pylori* stool antigen positivity rate of just 6.5% in our study is much lower than reports from population-based studies from even low *H. pylori* prevalence settings [35–37]. Stool antigen test has been reported to have good sensitivity and specificity for the detection of active *H. pylori* infection. However, bacterial load, exposure to antibiotic or PPI treatment, and requirements for proper handling of fecal samples, particularly in warmer settings, are known to reduce sensitivity considerably [17]. Thus, the low yield of our used antigen test in a known high *H. pylori* prevalence setting like Ethiopia casts some doubts on the diagnostic accuracy of the type of stool antigen test, which may also be linked to limitations in manufacturing quality, as the manufacturer did not provide any data for test validation.

As discussed, we have to assume that both test assays that were applied in this study have limitations in their validity to detect active infection. However, as they are the only diagnostic options readily available in Ethiopia, we tried to give a clearer picture of what a treating physician may face in terms of reported symptoms and constellations in the test results. We believe that the use of clinical data along with these tests may help improve treatment decisions in settings with limited options. Until more reliable and at the same time affordable diagnostic tests become available, such an approach may help to improve the balance between appropriate use of antibiotics and early treatment of *H. pylori* infection to prevent related adverse outcomes.

We performed a multivariable analysis with various assumptions to identify variables independently associated with either of the *H. pylori* tests. A logistic regression analysis has revealed that epigastric symptoms in the past three months and history of recent treatment with PPI were independently associated with *H. pylori* seropositivity. Although this may indicate that serology can be used as a screening test in individuals with symptoms of dyspepsia, its routine use as an indicator of active infection should be cautiously approached.

### 4.1. Strengths and Limitations of the Study

Our study involved a relatively large sample size involving rural and urban communities as well as children and adults. Besides, we tried to simulate the real-life situation by (1) selecting predominantly rural residents to adequately represent the population of the country and (2) using *H. pylori* tests used in routine clinical practice. However, the findings should be interpreted with caution due to the major limitations of the study. First of all, we did not compare these two tests against a gold-standard diagnostic test; therefore, our data does not confer any test validation. We took a pragmatic approach to compare the results of available laboratory tests with the presence of symptoms of dyspepsia as a proxy for *H. pylori* associated upper GI conditions. While this approach was used to simulate the real-life clinical scenario in Ethiopia, it is far from being ideal, as symptomatology by itself is hampered by being of low sensitivity and specificity for active *H. pylori* infection. Furthermore, the limited capacity of young children to accurately describe symptoms of dyspepsia might have also affected our findings. Moreover, the sensitivity and specificity of the particular kits we used for both tests were not available from the manufacturer, health authorities in Ethiopia, or previously published works.

## 5. Conclusion

Diagnosis of *H. pylori* infection with commercially available tests remains a challenge in Ethiopia. While stool antigen tests showed low yield, serology showed a better correlation with recent symptoms of dyspepsia. Relying on stool antigen tests as an indicator of active infection alone could miss patients with *H. pylori* infection that might need early treatment. At the same time, using serology tests for treatment decisions may lead to antibiotic overuse. With such limitations in validity of the available tests, proper diagnosis and early treatment of the infection and ensuring rational use of antibiotics remain a challenge for the treating physician. Thus, further research and development of cost-effective tests with better yield to detect active *H. pylori* infection in settings like Ethiopia are needed.

## Figures and Tables

**Table 1:** Background characteristic of study participants.

		Total	Serology, *N* (%)		Stool antigen, *N* (%)	
		(*N* = 1392), *N* (%)	Positive (*N* = 683)	Negative (*N* = 709)	Positive (*N* = 91)	Negative (*N* = 1301)
Age	1–5 years	344 (24.7)	101 (29.4)	243 (70.6)	37 (10.8)	307 (89.2)
	6–10 years	91 (6.5)	42 (46.2)	49 (53.8)	14 (15.4)	77 (84.6)
	11–19 years	292 (21.0)	156 (53.4)	136 (46.6)	16 (5.5)	276 (94.5)
	20–29 years	298 (21.4)	162 (54.4)	136 (45.6)	10 (3.4)	288 (96.6)
	≥30 years	367 (26.4)	222 (60.5)	145 (39.5)	14 (3.8)	353 (96.2)

Sex	Male	600 (43.1)	301 (50.2)	299 (49.8)	44 (7.3)	556 (92.7)
	Female	792 (56.9)	382 (48.2)	410 (51.8)	47 (5.9)	745 (94.1)

Residency	Urban	194 (13.9)	74 (38.1)	120 (61.9)	22 (11.3)	172 (88.7)
	Rural	1198 (86.1)	609 (50.8)	589 (49.2)	69 (5.8)	1129 (94.2)

Employment	Civil servant	61 (4.4)	35 (57.4)	26 (42.6)	1 (1.6)	60 (98.4)
	Student	303 (21.8)	166 (54.8)	137 (45.2)	19 (6.3)	284 (93.7)
	Farmer	379 (27.2)	240 (63.3)	139 (36.7)	17 (4.5)	362 (95.5)
	Unemployed	74 (5.3)	42 (56.8)	32 (43.2)	2 (2.7)	72 (97.3)
	Others	144 (10.3)	62 (43.1)	82 (56.9)	4 (2.8)	140 (97.2)
	Not applicable (children)	431 (31.0)	138 (32.0)	293 (68.0)	48 (11.1)	383 (88.9)

Education	No formal education	936 (67.2)	493 (52.7)	443 (47.3)	65 (6.9)	871 (93.1)
	Primary education	122 (8.8)	53 (43.4)	69 (56.6)	2 (1.6)	120 (98.4)
	Secondary education	98 (7.0)	56 (57.1)	42 (42.9)	5 (5.1)	93 (94.9)
	Tertiary completed	57 (4.1)	30 (52.6)	27 (47.4)	1 (1.8)	56 (98.2)
	Preschool children	179 (12.9)	51 (28.5)	128 (71.5)	18 (10.1)	161 (89.9)

**Table 2:** *H. pylori* serology and stool antigen test positivity among people with symptoms of dyspepsia.

		Total, *N* (%)	Serology			Stool antigen		
			Positive, *N* (%)	Negative, *N* (%)	*P*	Positive, *N* (%)	Negative, *N* (%)	*P*
Upper abdominal symptoms	Yes	681 (48.9)	398 (58.4)	283 (41.6)	<0.001^*∗*^	30 (4.4)	651 (95.6)	0.002^*∗*^
	No	711 (51.1)	285 (40.1)	426 (59.9)		61 (8.6)	650 (91.4)	
Epigastric pain	Yes	440 (31.6)	261 (59.3)	179 (40.7)	<0.001^*∗*^	18 (4.1)	422 (95.9)	0.014^*∗*^
	No	952 (68.4)	422 (44.3)	530 (55.7)		73 (7.7)	879 (92.3)	
Epigastric discomfort	Yes	592 (42.5)	352 (59.5)	240 (40.5)	<0.001^*∗*^	28 (4.7)	564 (95.3)	0.021^*∗*^
	No	800 (57.5)	331 (41.4)	469 (58.6)		63 (7.9)	737 (92.1)	
Epigastric burning	Yes	526 (37.8)	322 (61.2)	204 (38.8)	<0.001^*∗*^	19 (3.6)	507 (96.4)	0.001
	No	866 (62.2)	361 (41.7)	505 (58.3)		72 (8.3)	794 (91.7)	
Feeling full too long	Yes	379 (27.2)	218 (57.5)	161 (42.5)	<0.001^*∗*^	13 (3.4)	366 (96.6)	0.003^*∗*^
	No	1013 (72.8)	465 (45.9)	548 (54.1)		78 (7.7)	935 (92.3)	
Early satiety	Yes	411 (29.5)	244 (59.4)	167 (40.6)	<0.001^*∗*^	17 (4.1)	394 (95.9)	0.021^*∗*^
	No	981 (70.5)	439 (44.8)	542 (55.2)		74 (7.5)	907 (92.5)	
Heart burn	Yes	426 (30.6)	255 (59.9)	171 (40.1)	<0.001^*∗*^	21 (4.9)	405 (95.1)	0.107^*∗*^
	No	964 (69.4)	427 (44.3)	537 (55.7)		70 (7.3)	894 (92.7)	
Acid regurgitation	Yes	268 (19.3)	161 (60.1)	107 (39.9)	<0.001^*∗*^	12 (4.5)	256 (95.5)	0.168
	No	1124 (80.7)	522 (46.4)	602 (53.6)		79 (7.0)	1045 (93.0)	
Upper abdominal bloating	Yes	303 (21.8)	181 (59.7)	122 (40.3)	<0.001^*∗*^	11 (3.6)	292 (96.4)	0.018^*∗*^
	No	1089 (78.2)	502 (46.1)	587 (53.9)		80 (7.3)	1009 (92.3)	
Excessive belching	Yes	418 (30.0)	236 (56.5)	182 (43.5)	<0.001^*∗*^	19 (4.5)	399 (95.5)	0.048
	No	974 (70.4)	447 (45.9)	527 (54.1)		72 (7.4)	902 (92.6)	
Nausea	Yes	265 (19.0)	157 (59.2)	108 (40.8)	<0.001^*∗*^	17 (6.4)	248 (93.6)	0.923
	No	1127 (81.0)	526 (46.7)	601 (53.3)		74 (6.6)	1053 (93.4)	
Receiving treatment for dyspepsia in the last 30 days	Yes	181 (13.0)	115 (63.5)	66 (36.5)	<0.001^*∗*^	8 (4.4)	173 (95.6)	0.26
	No	1211 (87.0)	568 (46.9)	643 (53.1)		83 (6.9)	1128 (93.1)	
Previous diagnosis with stomach ulcer	Yes	68 (4.9)	43 (63.2)	25 (36.8)	<0.001^*∗*^	1 (1.5)	67 (98.5)	0.124
	No	1324 (95.1)	640 (48.3)	684 (51.7)		90 (6.8)	1134 (93.2)	
Seeking treatment for dyspepsia in the last 30 days	Yes	361 (25.9)	207 (57.3)	154 (42.7)	<0.001^*∗*^	15 (4.2)	346 (95.8)	0.035^*∗*^
	No	1031 (74.1)	476 (46.2)	555 (53.8)		76 (7.4)	955 (92.6)	

^*∗*^Statistically significant.

**Table 3:** Logistic regression analysis of clinical characteristics associated with *H. pylori* seropositivity.

	AOR	95% CI	*P*
Upper abdominal discomfort	1.130	0.692–1.845	0.626
Epigastric pain	0.747	0.473–1.180	0.211
Epigastric discomfort	1.515	0.960–2.391	0.074
Epigastric burning	1.929	1.278–2.911	0.002^*∗*^
Feeling full too long	0.703	0.477–1.035	0.074
Early satiety	1.219	0.837–1.777	0.302
Heart burn	1.264	0.865–1.848	0.226
Acid regurgitation	0.878	0.568–1.357	0.559
Upper abdominal bloating	1.077	0.750–1.547	0.689
Excessive belching	0.744	0.532–1.041	0.084
Nausea	1.219	0.886–1.678	0.224
Receiving treatment for dyspepsia in the last 30 days	1.509	1.045–2.178	0.028^*∗*^
Previous diagnosis with stomach ulcer	1.152	0.660–2.009	0.619
Seeking treatment for dyspepsia in the last 30 days	0.899	0.658–1.228	0.503

^*∗*^Statistically significant.

## Data Availability

All the data used to support the findings of this study can be obtained from the corresponding author.
